# Assessing the Prevalence and Determinants of Retinopathy of Prematurity in King Saud Medical City, Saudi Arabia

**DOI:** 10.7759/cureus.101577

**Published:** 2026-01-15

**Authors:** Ahmed Ghaly, Saleh A AlKhaldi, Mohammed A Radwan, Montaser Elmeqdad, Nabeel Alodaidan, Abdul Aziz Abdulsamad, Eiman Elbehery, Khader J. M. Abdul, Omnia M Sherif

**Affiliations:** 1 Neonatology, King Saud Medical City, Riyadh, SAU; 2 Research Center, King Fahad Medical City, Riyadh, SAU; 3 Ophthalmology, Faculty of Medicine, Cairo University, Cairo, EGY; 4 Ophthalmology, King Saud Medical City, Riyadh, SAU; 5 Pediatrics, King Saud Medical City, Riyadh, SAU

**Keywords:** epidemiology, oxygen inhalation therapy, premature birth, rop, saudi arabia, visual impairment

## Abstract

Purpose: This study aimed to examine the incidence, risk factors, and outcomes of preterm infants diagnosed with retinopathy of prematurity (ROP).

Methods: A retrospective cohort study was conducted involving infants with birth weight (BW) ≤1500 g or gestational age (GA) ≤32 weeks admitted to the neonatal intensive care unit at King Saud Medical City (KSMC) from December 2019 to December 2023.

Results: 593 infants were recruited; among them, 392 had available data. Of these, 93 (23.7%) infants had ROP, of which 17 (18.3%) were stage 3. The mean BW of infants diagnosed with ROP was 1265.4±324.1 g, while the mean GA at birth was 29.6±2.45 weeks. Significant correlation was found between ROP occurrence and young GA at birth, low BW, low Apgar score at 1 and 10 minutes, and long duration of oxygen therapy. After a medical records review, no subject had blindness.

Conclusion: The prevalence of ROP in this study is close to that reported in the region. Low BW and young GA were the most significant risk factors in the city. Controlling the amount and duration of oxygen therapy to the minimum necessary seems advisable.

## Introduction

Retinopathy of prematurity (ROP) is a main reason affecting vision in premature infants [1], and it was first diagnosed in 1942 [2]. The retinal vasculature develops by vasculogenesis and angiogenesis. At roughly 12 weeks of gestation, vasculogenesis begins as vascular precursor cells emerge from the hyaloid artery, which migrate peripherally to form the retinal arcades, while mesenchymal cells aggregate to develop vascular cords. It is generally completed by the 21st or 22nd week. At 17-18 weeks of gestation, retinal angiogenesis leads to the development of perifoveal, peripheral, and deep plexus vessels along with the capillary system in the fetal retina. It is generally completed around 36-40 weeks of gestation, when the superficial and deep retinal vessels reach the ora serrata [3], as vascular endothelial growth factor (VEGF) is essential for retinal vasculogenesis [4,5]. ROP is a process regulated by oxygen and develops in two phases. In the first phase, the increased oxygen content of the extrauterine environment leads to relative hyperoxia and subsequent vasoconstriction, halting normal vascular development. In the second phase, abnormal neovascularization occurs as a compensatory mechanism featuring VEGF upregulation [6-8]. VEGF is potentiated by insulin-like growth factor 1 (IGF-1), which is deficient in preterm infants. Optimal concentrations of VEGF and IGF-1 are critical for regular growth and development of several tissues, including eyes and blood vessels [9]. In fact, exposure to higher oxygen levels leads to vasoconstriction and ischemia, which in turn upregulates VEGF production by mesenchymal spindle cells and stimulates the creation of immature new vessels [10,11]. In addition, previous comparisons between preterm infants showed that erythropoietin level was indeed highly increased in the eyes of preterm infants diagnosed with ROP [12,13].

In 2010, it was estimated that ROP is responsible for mild-to-moderate visual impairment in more than 12,300 infants, and severe visual impairment in more than 20,000 as a total number worldwide [14]. Exposure to high oxygen levels, younger gestational age (GA), and lower birth weight (BW) are common risk factors of ROP. Further risk factors such as maternal diabetes mellitus, maternal smoking, premature rupture of membranes, chorioamnionitis, low Apgar score, respiratory distress syndrome, sepsis, hyperglycemia and insulin, and genetic factors have been postulated [15,16].

ROP is classified into five stages: stage 1 is characterized by the formation of a demarcation line, which progresses to stage 2, where it becomes a ridge. Stage 3 demonstrates fibrovascular retinal proliferation, while stages 4 and 5 feature partial retinal detachment and complete retinal separation, respectively [17].

International Classification of ROP (ICROP) has three retinal zones based on location: zone 1 is posterior, represents the least mature vascularization, and is defined as a circle centered around the optic disc with a radius double the distance between the fovea and the optic disc. Zone 2 is intermediate and is defined as a circle centered also around the optic disc with a radius equal to the distance between the optic disc and the nasal retinal periphery. Zone 3 is the residual crescent temporal of immature retina after the nasal part of the retina has developed complete vascularization [18-20]. Despite the relatively high prevalence of ROP and its complications, epidemiological data from Saudi Arabia are still scarce. Therefore, the primary objective of the present investigation is to examine the prevalence of ROP in premature infants in a tertiary Saudi hospital, and the secondary objective was to explore its risk factors, severity, and outcomes.

## Materials and methods

Study design and setting

This retrospective cohort study was conducted in the Neonatology Department of King Saud Medical City (KSMC), a major tertiary-care government hospital in Riyadh, Saudi Arabia. The department operates a Level III neonatal intensive care unit (NICU) with 40 ventilated beds and an additional 40 beds in the special care nursery. The study period extended from December 2019 to December 2023. All infants admitted to the NICU during the study period were screened for eligibility criteria, including BW ≤1500 g and/or GA ≤32 weeks. Infants were excluded if they had significant congenital anomalies, died before NICU discharge, or lacked complete ophthalmologic screening data.

Clinical evaluation and data collection

Data were extracted from the electronic medical records and the NICU clinical database, including demographic and perinatal characteristics such as sex, birth date, GA (weeks), BW (grams), mode of delivery, maternal risk factors (e.g., diabetes and hypertension), when available. Neonatal clinical data included Apgar scores, duration of supplemental oxygen therapy, duration of mechanical ventilation and non-invasive ventilation, sepsis (culture-proven or clinically diagnosed), necrotizing enterocolitis (NEC), classified using Bell’s criteria [21], intraventricular hemorrhage, graded by Papile classification [22], antenatal steroid exposure, postnatal steroid therapy, surfactant therapy, caffeine use, and other relevant neonatal comorbidities. Ophthalmologic assessment, including ROP screening, was performed by specialized ophthalmologists using standard international guidelines from the American Academy of Pediatrics screening recommendations [23].

Follow-up examinations were conducted at one-, two-, or three-week intervals according to ROP stage until complete peripheral vascularization is confirmed with or without treatment. At each examination, the presence or absence of ROP, ROP stage, and zone was documented based on ICROP [18-20], and plus disease is defined as vascular dilation and tortuosity within zone 1. Treatment was indicated when reaching stage 2 or 3 in zones 1 or 2 with plus disease. All cases received initial treatment with intravitreal injection of 0.025 mg anti-VEGF ranibizumab in one or two sessions, with indirect laser photocoagulation reserved for either non-responsive or recurrent cases after two injections, or persistent peripheral avascularity defined as persistent ROP of any stage or zone beyond 60 weeks of postmenstrual age [2]. The primary outcome was the development of any stage of ROP. Secondary outcomes included risk factors associated with ROP and severity (mild vs. severe ROP requiring treatment).

Statistical analysis

All analyses were performed using SPSS version 25 (IBM Corp., Armonk, NY, USA). Continuous variables were assessed for normality and reported as mean ± standard deviation. Categorical variables were presented as frequencies and percentages. Associations between risk factors and ROP were evaluated using the Chi-square test or Fisher’s exact test, as appropriate. Continuous variables were compared using the independent t-test when applicable. A p-value <0.05 was considered statistically significant.

Ethical considerations

Ethical approval was obtained from the Institutional Review Board of the Saudi Ministry of Health. The study adhered to the ethical principles of the Declaration of Helsinki, national guidelines for human research, and Good Clinical Practice (GCP) standards. All patient data were anonymized to maintain confidentiality.

## Results

**Figure 1:**
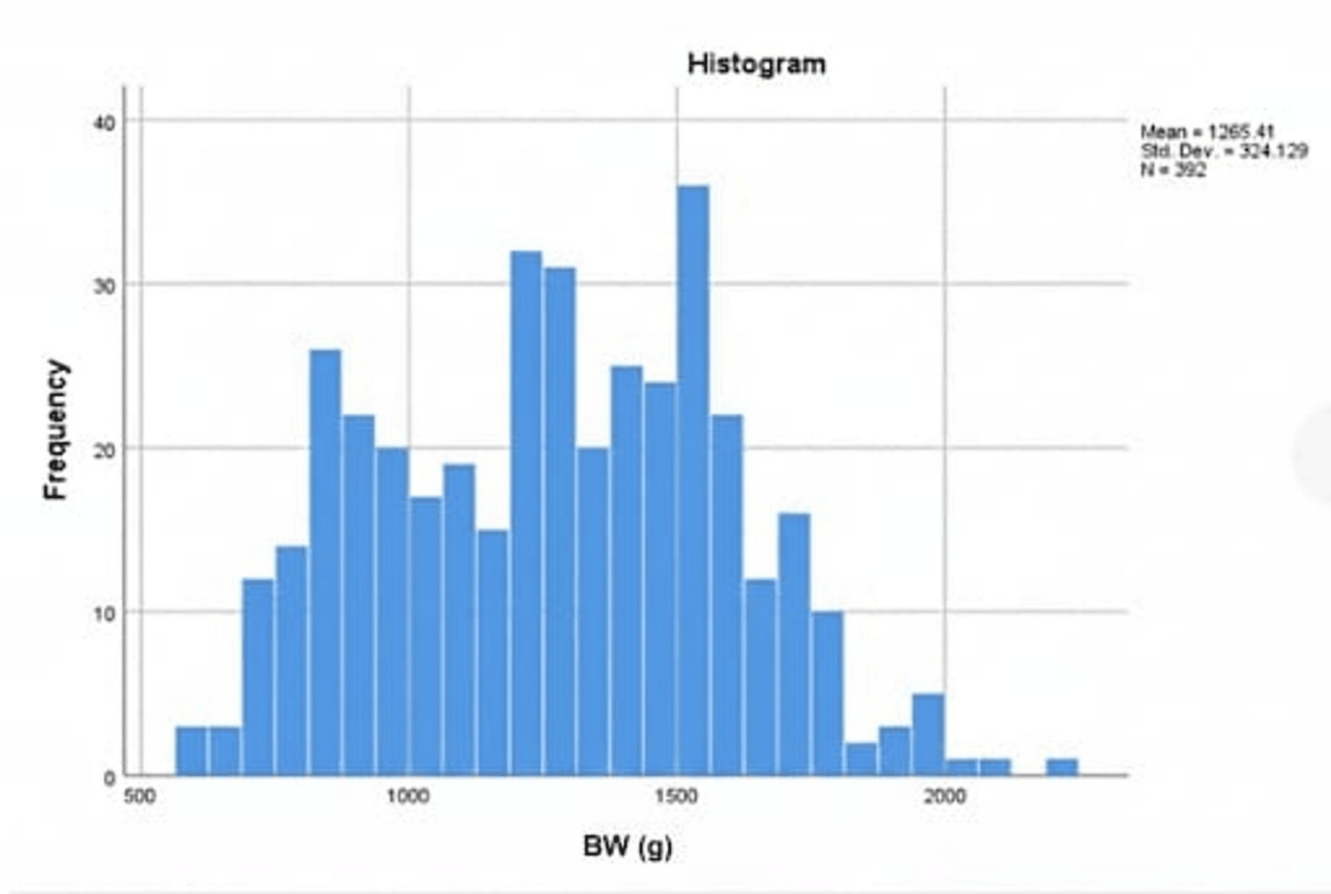
Mean BW at birth Figure 1 shows the frequency distribution of BW in grams, with great variation from 600 g to more than 2000 g and a higher frequency between 1000 g and 1500 g. BW, birth weight.

**Figure 2:**
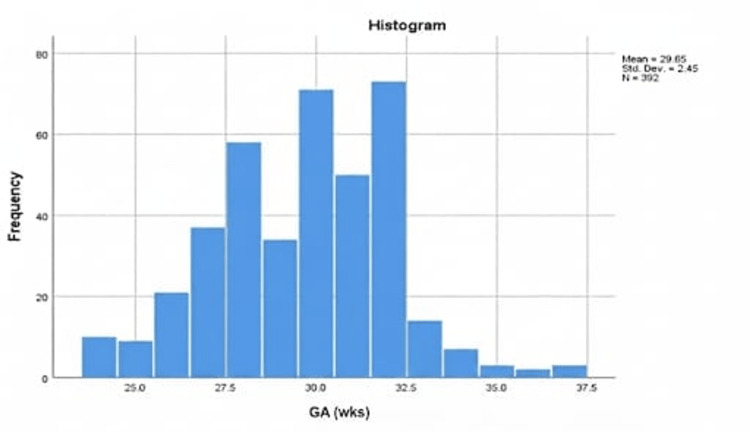
Mean GA at birth Figure 2 shows the frequency distribution of GA in weeks, showing a similarly great variation to that of BW, ranging from slightly older than 20 weeks to 37.5 weeks, with a higher frequency between 27.5 weeks and 32.5 weeks. BW, birth weight; GA, gestational age.

A total of 485 files have been reviewed. Of these, 10 incomplete files and 26 files belonging to deceased infants were excluded. Additionally, 57 cases were transferred to other hospitals before examination. Ultimately, 392 files were included in our study. Among the included cases, 183 (46.7%) infants were male, while 209 (53.3%) were female. Overall, 146 (37.2%) infants were delivered via normal spontaneous vaginal delivery, while 246 (62.8%) were born via cesarean section. The mean BW was 1265.4±324.1 g, with a range of 430-2280 g (Figure 1), while the mean GA at birth was 29.6±2.4 weeks, with a range of 24-37 weeks (Figure 2).

Of the infants, 314 (80.1%) were singletons, while 78 (19.9%) were born from multiple pregnancies. Additionally, ROP was diagnosed in 93 (23.7%) infants, among which 4 (4.3%) had stage 2 and 17 (18.3%) had stage 3 (Table 1).

**Table 1:** Distribution of ROP among the infants Table 1 shows the percentage of different stages of ROP with or without plus disease, showing that all stage 1 and most of stage 2 are without plus, and all stage 3 are with plus disease. ROP, retinopathy of prematurity.

ROP stage	1	2	3
Without plus	49 (52.7%)	23 (24.7%)	0
With plus	0	4 (4.3%)	17 (18.3%)

**Figure 3:**
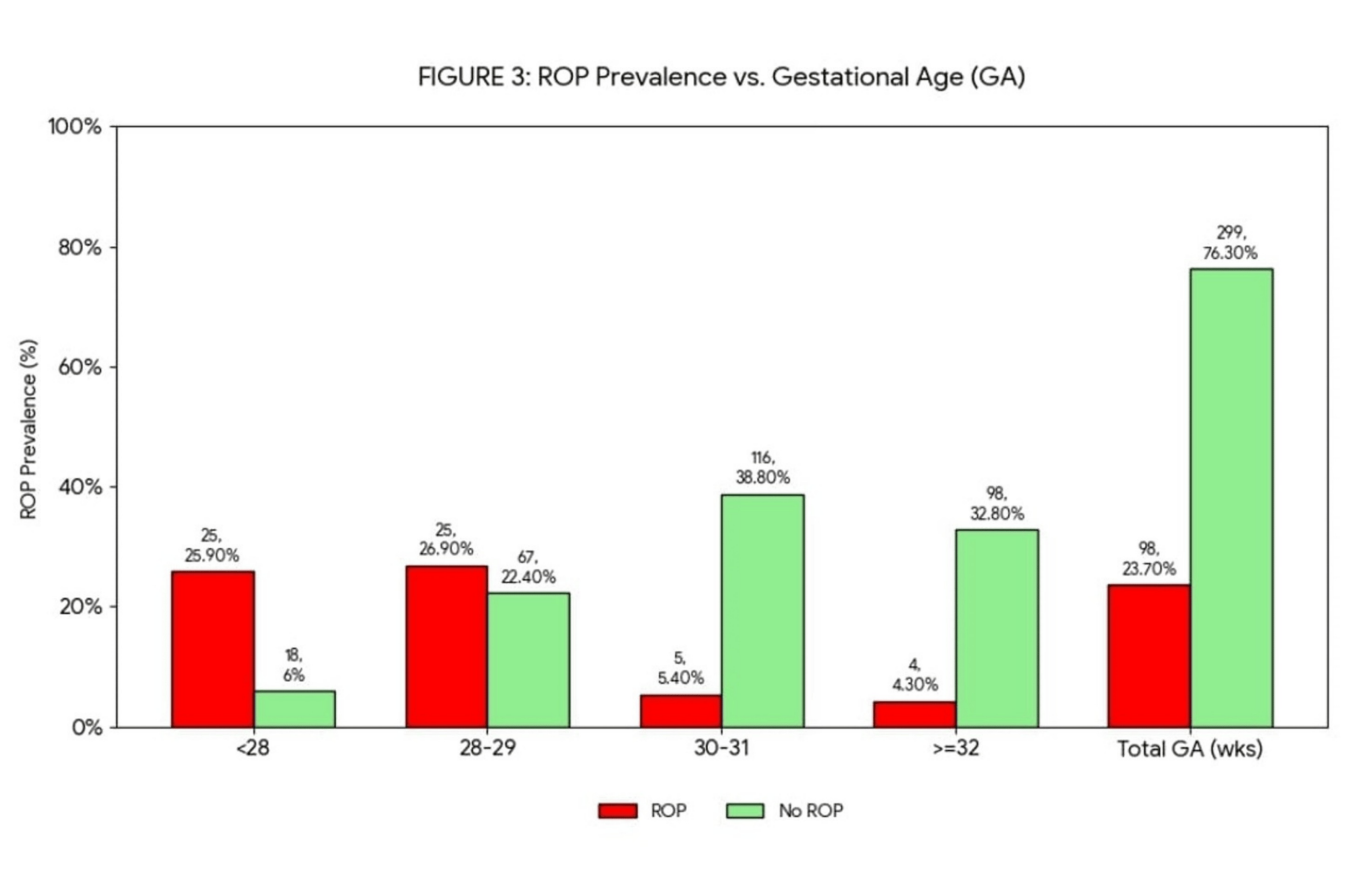
BW in subjects with ROP vs. without ROP Figure 3 shows the percentage of ROP incidence in relation to GA, with a significant increase at early ages. BW, body weight; GA, gestational age; ROP, retinopathy of prematurity.

The mean GA of infants with ROP was 27±2.4 weeks, while the mean BW was 938.4±257.9 g (median: 907.5 g; range: 430-2280 g). Overall, 131 infants (60%) had bilateral involvement, 42 (19%) were affected unilaterally on the left side, and 45 (21%) unilaterally on the right side. Three subjects (1.48%) had threshold ROP, which required treatment (i.e., stage 3, zone 1 or 2 with plus disease and at least five contiguous or eight cumulative hours of involvement). According to our comparative analysis, subjects with ROP were likely to have a BW <1000 g and a GA <28 weeks compared with healthy subjects (p<0.05; Figure 3).

Risk factors for ROP included GA at birth (p<0.05), BW (p<0.05), Apgar score at 1 min after birth (p>0.05), duration of oxygen therapy (p<0.001), and duration of mechanical ventilation (p <0.001), which are statistically significant. By contrast, the mother’s age at the time of delivery (p= 0.703), the number of infants a mother gave birth to (p=0.051), and the Apgar scores at 5 minutes (p=0.190) and 10 minutes (p=0.280) did not reach statistical significance. BW <1500 g at birth was the most significant risk factor for ROP (odds ratio (OR)=11.7, 95% CI: 1.08-126.03, p<0.05), followed by GA at birth <32 weeks (OR=3.34, 95% CI: 1.21-9.15, p<0.05).

Three patients had a laser treatment while 12 received an anti‑VEGF treatment, most commonly ranibizumab. Based on the chart review, no infants had an unfavorable structural outcome at least three months after discharge. Moreover, five patients with ROP had a GA >32 weeks, including one at 35 weeks, two at 34 weeks, and two at 33 weeks. Additionally, two subjects with BW >1500 g were found to have ROP (Table 2).

**Table 2:** Multiple backward logistic regression analysis of the variables Table 2 shows how most of the other systemic variables are significantly linked to the incidence of ROP occurrence. *Highly statistically significant. NEC, necrotizing enterocolitis; PDA, patent ductus arteriosus; RBC, red blood cell; ROP, retinopathy of prematurity.

Variable	Non-ROP (n=299)	ROP (n=93)	p-value	Univariate odds ratio (95% CI)	Adjusted odds ratio (95% CI)
GA in weeks (mean±SD)	30.4±1.97	27.0±1.93	0.0000*	0.395 (0.32–0.48)	-
BW in grams (mean±SD)	1370.1±270.6	923.9±241.5	0.0000*	28.6 (10.5–77.3)	-
Gender – male (n, %)	141 (47.2%)	42 (45.2%)	0.736	-	-
Multifetal gestation (n, %)	61 (20.4%)	18 (19.4%)	0.8339	-	-
Delivery – cesarean (n, %)	195 (65.2%)	51 (54.8%)	0.1716	-	-
Apgar score at 1st minute (n, %) – <6	90 (57.3%)	67 (42.7%)	<0.0001*	1.61 (1.4–1.8)	-
Apgar score at 1st minute (n, %) – ≥6	205 (89.1%)	25 (10.9%)	<0.0001*	1	-
Surfactant therapy – yes (n, %)	125 (41.8%)	92 (98.9%)	<0.0001*	128 (17.6–931)	-
Postnatal steroids (n, %)	4 (1.3%)	28 (30.1%)	<0.0001*	0.03 (0.01–0.09)	-
Packed RBCs (n, %)	85 (28.4%)	91 (97.8%)	<0.0001*	114.5 (27.5–475.5)	-
Sepsis – yes (n, %)	94 (31.4%)	92 (98.9%)	<0.0001*	200.6 (27.5–1461.4)	25.8 (2.1–308.7)
PDA (n, %)	64 (21.4%)	46 (49.5%)	<0.0001*	3.5 (2.1–5.8)	4.4 (1.5–12.9)
Intraventricular hemorrhage (n, %)	45 (15.1%)	71 (76.3%)	<0.0001*	18.2 (10.2–32.3)	-
Seizure – yes (n, %)	14 (4.7%)	27 (29%)	<0.0001*	8.3 (4.1–16.7)	-
NEC – yes (n, %)	35 (11.7%)	42 (45.2%)	<0.0001*	6.2 (3.6–10.6)	-
Hyperglycemia (n, %)	4 (1.3%)	43 (46.2%)	<0.0001*	63 (21.8–184.4)	36.7 (4.6–289)

## Discussion

The examining ophthalmologist recommends follow-up examinations based on retinal findings categorized by the ICROP [6]. In Saudi Arabia, the local regulations recommend screening premature infants with GA at birth <32 weeks or a BW <1500 g [14]. Additionally, infants with other comorbidities such as oxygen therapy, NEC, septicemia, and surfactant use receive more attention. Every premature newborn underwent a fully dilated fundus examination at the postmenstrual age of 31 weeks and at the chronological (postnatal) age of 4-6 weeks, whichever happened later, using a scleral depressor and a lid speculum.

In babies at risk, the uniformly reported incidence of ROP varies from 29% to 68% [7,8,15-17]. Around 23.7% of the patients in this study had a diagnosis of ROP, which is comparable to what was reported in a previous study [7], but significantly lower than the findings of another study [8]. The greater sample size, the longer review period (2010-2014), and the agreement with one of the local studies could all be responsible for this discrepancy. According to a study published in 2012, no significant difference was found between males and females diagnosed with ROP or the number of babies born [18].

The role of certain risk factors for ROP, such as low BW, young GA at birth, low Apgar score at 1 minute, and long oxygen-therapy duration, has been found to be significant in most existing studies [7-11,15-18]. The incidence of ROP in this study was found to be associated with the duration of oxygen therapy rather than the amount, and our NICU adheres to a conservative oxygen saturation policy (i.e., 88%-92%).

It is interesting to note that five patients with ROP had a GA >32 weeks during the chart analysis; one of them had a GA of 35 weeks, two of 34 weeks, and two of 33 weeks. Furthermore, two infants had ROP with a BW of more than 1500 g, similar to previous publications [7]. These findings suggest that the local screening regulations in the kingdom may require revision to include patients with GA >32 weeks and BW >1500 g to reduce the expected extra burden on the health system. More widely spaced examinations are recommended as children grow older, between 45 and 55 post-menstrual age, where less frequent change of reactivation and recurrence has been noticed.

With advancements in ROP treatment, such as laser, prognostic improvements are expected. Although blindness caused by ROP is a serious issue globally, with at least 50,000 infants affected by ROP-induced blindness [19-25], none of the patients included in the current study developed blindness. This could be attributed to careful screening and proper management, which are key factors in preventing blindness in these patients.

This study has a number of limitations worth mentioning. It is a retrospective, single-center study. Furthermore, the follow-up period was relatively short. Another point worth mentioning is the excluded infants (transferred or deceased), which may affect generalizability. 

## Conclusions

The results of the current study are comparable to the literature. Low BW and young GA were identified as the most significant risk factors. Although blindness is a relatively common consequence of ROP, it was not observed in the present study subjects. Future studies with a greater sample size and multicenter settings are recommended for conclusive findings.
